# Animal Models of Alcoholic Liver Disease—Focus on the Intragastric Feeding Model

**Published:** 2003

**Authors:** Amin A. Nanji, Samuel W. French

**Affiliations:** Amin A. Nanji, M.D., is a professor in the Department of Pathology at the University of Pennsylvania, Philadelphia. Samuel W. French, M.D., is chief of the Division of Anatomic Pathology and a professor in the Department of Pathology at the Harbor–UCLA Medical Center, Los Angeles, California

**Keywords:** animal model, alcoholic liver disorder, disease course, disease severity, gene regulation, biochemical mechanism, genetics and heredity, nutrition, oxygen, smoking, endotoxin, hypoxia

## Abstract

The use of animal models has contributed to greater understanding of how alcoholic liver disease (ALD) develops, and of how the severity of liver injury is influenced by factors other than alcohol, such as nutrition, oxygen deprivation (as occurs with sleep apnea or smoking), and gene regulation. This article focuses on the use of one animal model in particular, the intragastric feeding model in rats. This model allows scientists to rigorously control an animal’s consumption of both alcohol and dietary nutrients and is providing important information on the mechanisms of injury of alcoholic liver disease.

Ideally, research using animals to examine a disease that occurs in humans will re-create in the animals all of the human disease characteristics. For example, chimpanzees experimentally infected with the hepatitis C virus develop abnormalities in the liver that closely resemble those observed in humans who have acquired the viral infection naturally. In the case of alcoholic liver disease (ALD), no such comprehensive animal model currently exists (Hall et al. 2001). Researchers have not been able to develop this model for reasons that are not understood as yet, but which probably involve genetic differences between humans and other animals.^1^

In the absence of a comprehensive model, ALD studies using animals are designed to answer specific questions about different aspects of the disease, usually addressing only one or two experimental variables at a time while holding others constant. This is the same approach that scientists use in general; however, in animal studies, maintaining experimental control is especially difficult, because changes that are experimentally introduced in the animal are modified by compensating biological mechanisms, such as changes in blood flow and metabolic rate, that maintain internal equilibrium (i.e., homeostasis). Despite this challenge, addressing only one or two experimental variables at a time enables researchers to isolate and investigate mechanisms that cause the disease or affect its severity.

Our goal here is not to address all of the animal models of liver disease, such as the Lieber–DiCarli model and the Lindros model, as this has been done in detail elsewhere. (See Hall et al. 2001 for a review.) Instead, we describe one animal model that is being used to solve specific questions related to the mechanisms of injury that lead to ALD, outline questions that have been addressed using this model, and briefly summarize the results of these investigations.

## The Intragastric Feeding Model

Because alcohol normally displaces other nutrients as a source of dietary calories, any animal model of ALD must rigorously control the intake of both alcohol and other nutrients. In rat and mouse models, this control can be accomplished best by feeding alcohol and food in liquid form through a tube (called a cannula) inserted permanently into the stomach (French et al. 1984). Under this regimen, known as the intragastric feeding model, animals can be given alcohol for 6 months or longer, and higher blood alcohol levels can be maintained than with other feeding regimens. In a procedure known as isocaloric pair-feeding, control animals receive the same diet, with glucose calories substituted for alcohol calories. Animals in both the experimental and control groups are fed continuously so that their nutrition can be compared precisely and simultaneously. Because urinary alcohol levels correlate closely with blood alcohol levels, the intragastric feeding model permits researchers to monitor animals’ alcohol levels daily without having to obtain blood. To determine how alcohol-induced liver injury develops, researchers can test parameters at regular intervals (Nanji et al. 1989). For instance, in the intragastric feeding model of ALD in rats, the progression of liver disease can be followed by examining liver biopsies taken monthly over a 6-month period (Nanji et al. 1989).

As with other models, the intragastric feeding model also can be combined with other experimental methods, including cell culture research (in which liver cells from alcohol-fed animals are extracted and examined in isolation) and research with genetically altered animals.

In a variation on the intragastric feeding model that is used for alcohol research with primates and specially bred small pigs (i.e., micropigs) as well as rodents (Jarvelainen and Lindros 2002; French et al. 1995), alcohol—but not food— is administered through a tube in the stomach. However, because animals are fed on demand in this model, the method does not permit investigators to control nutrition.

## Animal Models Are Designed to Address Specific Questions

### How Do Alcohol and Acetaldehyde Directly Affect the Liver?

Alcohol accelerates the breakdown of chemicals called methyl donors that help the body absorb fats (Tsukamoto and Lu 2001). If this breakdown leads to a deficiency of methyl donors, fats can accumulate in the liver, leading to liver damage. But if animals are fed alcohol together with an adequate diet that includes saturated fats and supplements such as choline, methionine, and S-adenosylmethionine, which compensate for the alcohol-induced breakdown of methyl donors, liver damage does not occur. (See “Macronutrients,” below, for further discussion of the role of different kinds of fats in ALD.) This finding suggests that neither alcohol nor its metabolite acetaldehyde is directly toxic to the liver, but this has not been firmly substantiated by the evidence. Although protein adducts of acetaldehyde form in the liver, the functional significance of this phenomenon has not yet been established.

### How Does Nutrition Affect ALD?

Experiments using the intragastric feeding model have clearly demonstrated that nutrition plays a crucial role in ALD by promoting normal metabolic processes in the liver or working in conjunction with alcohol to disrupt them.

#### Macronutrients^2^ (Carbohydrates, Proteins, Fats)

Research in rats has shown that dietary carbohydrates help avert alcohol-induced liver injury. Drinking high doses of alcohol for long periods of time increases the activity of an enzyme called cytochrome P450 2E1 (also known as CYP2E1), which triggers a cascade of events that cause liver injury. Among other things, this cascade involves the formation of highly reactive, oxygen-containing molecules (i.e., oxygen radicals) that can damage cells and tissues through a variety of mechanisms. The alcohol-induced accumulation of oxygen radicals and/or depletion of molecules that normally eliminate oxygen radicals (i.e., antioxidants) results in a state known as oxidative stress. (Other pathways in addition to CYP2E1 are primarily responsible for producing harmful oxygen radicals. For more information on the role of oxygen radicals and oxidative stress in the development of ALD, see the article by Wu and Cederbaum in this issue. For additional information about how alcohol use can lead to oxidative stress and subsequent liver injury, see the sidebar.)

**Alcohol Use, Oxidative Stress, and Liver Damage**

Most alcohol is broken down (i.e., metabolized) in the liver through a series of chemical reactions, known as oxidation reactions, which involve hydrogen and oxygen atoms. In the predominant biological pathway for alcohol metabolism, called the alcohol dehydrogenase pathway, the enzyme alcohol dehydrogenase (ADH) converts alcohol to a toxic intermediate substance, acetaldehyde, by removing two atoms of hydrogen from each alcohol molecule. Then a second enzyme, aldehyde dehydrogenase (ALDH), quickly converts acetaldehyde to acetate by again removing hydrogen and adding oxygen.

A second pathway of alcohol metabolism, the microsomal ethanol-oxidizing system (MEOS), is activated by long-term heavy alcohol consumption. The MEOS pathway involves the enzyme cytochrome P450 2E1, or CYP2E1, which (like ADH in the alcohol dehydrogenase pathway) strips hydrogen away from alcohol to produce acetaldehyde. In both of these pathways—but more markedly in the MEOS pathway—oxidation reactions spawn highly unstable molecules called free oxygen radicals. Examples of these include superoxide (O_2_^•^), hydrogen peroxide (H_2_O_2_), and hydroxyl radicals (^•^OH).

Normally, the body deploys molecules called antioxidants (for example, vitamin E, vitamin C, and glutathione, or GSH) to clear oxygen radicals from the liver. However, heavy alcohol use not only heightens the production of oxygen radicals but also depletes the body’s supply of antioxidants, creating an imbalance between oxygen radicals and antioxidants. This imbalance, known as oxidative stress, damages liver cell membranes as well as the cells’ mitochondria, their primary site of energy production. When oxidative stress is chronic, it contributes to cell death (i.e., necrosis) and liver tissue damage (i.e., fibrosis). In addition to its direct effects on the liver, oxidative stress appears to stimulate autoimmune reactions that further damage liver cells (Albano et al. 1998).

—Amin A. Nanji and Samuel W. French

ReferenceAlbanoEFrenchSIngelman-SundbergMCytochrome P450 2E1, hydroxyethyl radicals, and immune reactions associated with alcoholic liver injuryAlcoholism: Clinical and Experimental Research227407421998

Perhaps the most convincing data that oxidative stress contributes to alcohol-induced liver injury comes from studies using the intragastric feeding model of alcohol administration (Caro and Cederbaum 2004). In these studies, alcohol enhanced key mechanisms that lead to the destruction of liver cells (such as lipid peroxidation, protein carbonyl formation, and the formation of the 1-hydroxyl ethyl radical). In addition, alcohol decreased the body’s natural antioxidant defense.

High levels of dextrose in the diet prevent alcohol from stimulating the CYP2E1 enzyme’s activity. Experiments with alcohol-fed rats confirm that in animals with a low percentage of dietary calories derived from dextrose, blood alcohol levels remain high and liver injury increases, compared with alcohol-fed rats whose diets contain more dextrose (Jarvelainen and Lindros 2002).

Like dextrose, protein also helps keep ALD in check. In one investigation using the intragastric feeding model, rats fed alcohol and a low-protein, high-fat diet for 6 months had more severe liver injury, including cell death (centrilobular necrosis) and scar tissue development (fibrosis), than did rats who were fed the same diet without alcohol (French et al. 1988). A lesser degree of liver injury was seen if the diet contained adequate amounts of protein (French 1993).

Finally, as mentioned above, research using the intragastric feeding model has revealed that the type of dietary fat ingested by animals is a major factor in alcohol-induced liver damage (French 1993). Polyunsaturated fatty acids, such as those found in corn oil and other vegetable oils, promote destructive CYP2E1 activity by generating free radicals, which attack polyunsaturated lipids (a process known as lipid peroxidation) when alcohol is fed (French 1993), thereby altering the integrity of liver proteins.

In an experiment designed to examine this process, rats fed alcohol and saturated beef fat (i.e., tallow) were pair-fed with rats fed the same diet plus 2.5 percent linoleic acid (French 1993). Linoleic acid is a polyunsaturated fatty acid that the liver converts to arachidonic acid, a primary target for lipid peroxidation. (Lard, which can contribute to alcohol-induced liver injury, also contains 2.5 percent linoleic acid.) Rats fed alcohol and tallow plus linoleic acid developed liver injury after 6 months, whereas rats fed only alcohol and tallow did not (French 1993).

#### Micronutrients^3^ (Folate, Iron)

Animal models have revealed that—like carbohydrates, protein, and fat—micronutrients such as folate and iron also affect animals’ likelihood of developing ALD. For example, when micropigs were fed alcohol and a folate-deficient diet on demand for 14 weeks, they developed severe liver injury and an alteration in an essential amino acid (methionine) that prevents the harmful buildup of fats in the liver. Both methionine and folate also play a role in the availability of S-adenosylmethionine, which, in turn, is vital to many biochemical processes in the body that are essential for good health. Liver injury did not occur in animals fed alcohol and a folate-supplemented diet (Halstead et al. 2002).

Conversely, when alcohol-fed rats were given a small increase in dietary iron for 16 weeks, liver damage was markedly worse than it was in rats given the extra iron but no alcohol (Tsukamoto et al. 1995). Some animals developed cirrhosis, probably because iron increases the rate at which oxidative stress occurs.

### What Causes Liver Scarring in ALD?

Star-shaped cells called stellate cells, located in the walls of blood vessels in the liver, are the source of collagen, a protein that provides the basis for bone, cartilage, tendon, and other connective tissues, as well as for enzymes (such as proteases) that remove that collagen. Liver scarring occurs when the stellate cells produce more collagen than the proteases can remove. Examinations of cultured stellate cells isolated from the livers of alcohol-fed rats showed increased activation and collagen synthesis (French 2002). Although there also is an increase in protease synthesis, with time this becomes relatively diminished. Further, a diet rich in polyunsaturated fatty acids and alcohol amplified the effect (Tsukamoto and Lu 2001). Despite the value of tissue-cultured cells, an improved model for studying stellate cell activation in living animals is needed. Cell culture models do not take into account a live animal’s ability to maintain homeostasis, and these homeostatic mechanisms likely modify tissue responses to injury.

### What Are the Benefits of Supplementing the Diet With Polyenylphosphatidylcholine (PPC)?

Research with baboons has shown that providing alcohol as part of an on-demand liquid diet for several years leads to alcoholic cirrhosis in some animals. This outcome can be prevented, however, by supplementing the animals’ diet with polyenylphosphatidylcholine, or PPC (Lieber et al. 1997; Tsukamoto and Lu 2001). PPC is a phospholipid that has two molecules of linoleic acid. This phospholipid (used as a liver balm in Europe) acts as an antioxidant and inhibits collagen production by stellate cells in tissue culture.

Following this observation in baboons, researchers have investigated whether PPC might prevent liver disease in rats fed alcohol as part of an on-demand diet (the Lieber–DeCarli liquid diet) (Tsukamoto and Lu 2001). In cells isolated from rats’ livers and supplemented with PPC in cell culture, PPC had several beneficial effects, including reducing collagen production and acting as an antioxidant in a manner similar to vitamin E’s antioxidizing action. Studies of intact livers have shown that giving alcohol-fed animals PPC (1) enhanced the incorporation of the cell nutrient phosphatidylcholine (PC) into cellular membranes and (2) limited alcohol-induced increase in CYP2E1 activity, thus curbing the production of free oxygen radicals that lead to oxidative stress. PPC also suppressed the formation of fragmented dead cells, known as apoptotic bodies, in the liver (Tsukamoto and Lu 2001).

This research is a departure from the intragastric feeding model, in that animals received alcohol and food on demand (that is, nutrition and alcohol ingestion were not under the control of the investigator but rather depended on the will of the experimental animal).

### How Does Endotoxin Affect ALD?

Alcohol can cause bacterial cell wall material (i.e., endotoxin) to leak from the intestine and enter the blood, ultimately leading to oxidative stress and consequent liver damage. In the intragastric feeding model, rats’ endotoxin levels were highest when blood alcohol levels were at their peak (French 2001). Alcohol appears to sensitize the liver to endotoxin: Liver damage was much greater when rats were fed alcohol and endotoxin than when they were given alcohol alone (Tsukamoto and Lu 2001). (For more information on the role of endotoxin in the development of ALD, see the article by Wheeler in this issue.)

The body’s toxic response to endotoxin involves both the flat cells (endothelial cells) that line the vessel structures (or sinusoids) inside the liver as well as the immune system cells, called Kupffer cells, which are specific to the liver.

Endothelial cells bind circulating white blood cells, and the white cells then release toxic proteases and plug up cavities in the sinusoids, preventing blood flow within the sinusoids.

Endotoxin and alcohol stimulate Kupffer cells, as intragastric mouse and rat models have shown. Endotoxin binds to Kupffer cell receptors, initiating a cascade of events that lead to the release of a chemical messenger (i.e., cytokine) called tumor necrosis factor alpha (TNF–α). This molecule attracts immune cells to the liver and triggers the release of free oxygen radicals by Kupffer cells, leading to liver injury. In the rat model, antibodies to TNF–α, injected intravenously, markedly reduced alcohol-induced liver damage (French 2000). In the mouse model, alcohol-fed mice that genetically were deficient in receptors that bind TNF–α were protected from alcohol-induced liver injury (Crews 2001). (These findings demonstrate that genetically altered mice, fed alcohol intragastrically, can be a powerful tool for investigating ALD [Crews 2001].)

Studies of the role of endotoxin in liver injury highlight the importance of intestinal bacteria in early alcoholic liver disease and indicate two main ways to prevent experimental endotoxin-induced liver injury (French 2000). One method is to administer gadolinium chloride, a rare metal that eliminates Kupffer cells from the liver. The other is to inhibit the formation of endotoxin in the intestine, either by replacing native bacteria with nontoxic bacteria or by giving antibiotics (Enomoto et al. 2001).

### What Is the Role of Hypoxia in ALD?

The intragastric feeding model also has been used to study the effect of low levels of oxygen (O_2_) in the blood (hypoxia) on alcohol-induced liver injury in rats (Bardag-Gorce et al. 2002). Research using this model has shown that alcohol-fed rats subjected to diminished oxygen (6 percent O_2_ for 5 hours) experienced increased cell death (i.e., necrosis) in the centrilobular hepatocytes of the liver, compared with rats breathing the same air who were not given alcohol (French et al. 1984). The increased susceptibility to hypoxia caused by alcohol is thought to result from the increased metabolic rate and consequent increased demand for O_2_ when alcohol is metabolized. Results of this research suggest that the combination of alcohol ingestion and sleep apnea could damage the liver.

Similarly, rats fed alcohol intragastrically while breathing carbon monoxide (CO) for 4 months—a treatment that simulates smoking—showed evidence of increased liver injury. (CO reacts with hemoglobin in the blood to form carboxyhemoglobin, rendering hemoglobin incapable of transporting oxygen to tissues. The resulting decrease in oxygen delivery to tissues leads to tissue hypoxia.) These results suggest that the combination of alcohol ingestion and smoking may cause liver damage.

Research using the intragastric feeding model in rats indicates that hypoxic liver injury occurs when alcohol levels are at their peak. (Even though alcohol is administered continuously in this model, rats’ blood and urine alcohol levels fluctuate, peaking every 6 to 10 days.) Bardag-Gorce and colleagues (2002) observed several indicators of liver hypoxia when urine alcohol levels were high, and these symptoms were reversed when alcohol decreased to low levels. The results indicate that high blood alcohol levels, as seen in binge drinking, may injure the liver.

The finding that the effects of hypoxia can be blocked by administering an antithyroid drug or by cutting the pituitary stalk indicates that these effects may be driven by the cycling of thyroid hormone (French 2001). When thyroid hormone levels were high, the metabolic rate (that is, the rate of O_2_ consumption) and the rate of alcohol elimination were high. Consequently, blood alcohol levels were low. When thyroid hormone was blocked, this chain of events was interrupted. These studies indicate that there likely is a hormonal component to alcohol-induced liver injury.

### What Is the Role of Gene Regulation in the Development of ALD?

Processes leading to ALD are to some extent regulated by specific genes, as indicated by some of the research already mentioned (e.g., the study of alcohol-fed mice deficient in TNF–α receptors [Kono et al. 2001]). (See the table for a summary of genes whose involvement in ALD has received research attention.) A powerful tool for investigating gene regulation in ALD is the use of animals with genetic mutations that suppress the functioning of certain genes (that is, these genes are “knocked out”).

To better understand the processes that lead to liver disease, scientists have adapted the intragastric protocol for use in these gene-altered mouse models. For example, knockout animals have been used to study the role that alcohol-induced CYP2E1 plays in ALD. Research has shown that knockout mice, which had been specifically bred to be deficient in this enzyme, developed the same degree of liver injury as did normal, nonmutated mice, indicating that CYP2E1 may not have a central role in alcohol-induced liver injury. On the other hand, researchers (Chen and Cederbaum 1998) using a liver cell model showed that high levels of CYP2E1 (i.e., overexpressed CYP2E1) do indeed have a direct and toxic effect on liver cells, even in the absence of alcohol. Other scientists used the same knockout model cited above to show that when the CYP2E1 gene is overexpressed it causes alcohol to inhibit a key component involved in breaking down proteins, the 26S proteasome (Bardag-Gorce et al. 2000), which may account for some of the liver damage caused by alcohol. Alcohol-fed mice genetically altered to overexpress CYP2E1 gene activity (that is, transgenic mice) also showed greater liver injury than did normal (wild) mice fed alcohol (Morgan et al. 2002). Clearly, more research is needed to understand the mechanisms behind ALD. The apparent contradictions on the importance of CYP2E1 in ALD illustrate the difficulty involved in teasing apart the complex roles that specific genes and their products play in liver injury.

**Table:** Gene Alterations That Affect Risk for ALD

Gene	Effect on Liver Injury	Reference
CYP2E1 (knock-out)	Gave no protection	Crews 2001*
CYP2E1 (knock-out)	Prevented inhibition of the proteasome	Bardag-Gorce et al. 2002
CYP2E1 (transgenic)^1^	Increased liver injury	Morgan et al. 2002
TNF RI (knock-out)	Protected liver	Crews 2001*
TNF RII (knock-out)	Gave no protection	Crews 2001*
NADPH Oxidase (knock-out)	Protected liver	Crews 2001*
CD14 (knock-out)	Protected liver	Crews 2001*
TLR4 (knock-out)	Protected liver	Crews 2001*
ICAM (knock-out)	Protected liver	Crews 2001*
CuZnSOD (dismutation)^2^	Protected liver	Crews 2001*
MnSOD (dismutation)	Protected liver	Crews 2001*

NOTE: CYP2E1 = cytochrome P450 2E1; TNF RI and II = tumor necrosis factor receptors I and II; NADH = reduced nicatinamide adenine dinucleotide; CD14 is an endotoxin receptor; TLR4 is a toll receptor that responds to endotoxin together with CD14; ICAM = cell adhesion molecule binding leukocytes; CuZnSOD = copper zinc superoxide dismutase; MnSOD = manganese superoxide dismutase.

* Studies reviewed in this article were done in Ronald Thurman’s laboratory at the University of North Carolina Alcohol Research Center.

1 The term “transgenic” means that a gene is added to the egg (ovum) before the embryo forms.

2 Dismutation is an enyzme process that results simultaneously in oxidation and reduction–—for example, a superoxide radical is converted to H_2_O_2_ (hydrogen peroxide).

## Summary

Animal models such as the intragastric feeding model have afforded valuable insight into the development and progression of alcohol-induced liver injury. Lacking a single animal model that mimics ALD as it occurs in humans, researchers have used animal models to separately address specific questions about the disease, such as how ALD is affected by nutritional factors, dietary supplementation, endotoxin, and oxygen deprivation; the mechanisms by which liver scarring occurs; and the ways in which gene expression affects the early development of ALD. Research into all these questions has shed light on the extent to which alcohol indirectly affects the development of ALD. As a body, the research indicates that both genetic and nutritional factors strongly influence the development and progress of ALD.

## Footnotes

1 One possible difference is in the DNA sequence of a protein called ubiquitin, which is essential for the formation of abnormal structures called Mallory bodies in the liver cells of patients with ALD. The DNA for ubiquitin in rats has a different sequence, so that rat liver cells cannot form Mallory bodies in response to excess alcohol ingestion.

2 Macronutrients are those nutrients—such as carbohydrates, fats, and proteins—that are required in greatest amounts.

3 A micronutrient is a dietary substance, such as a vitamin or mineral, that is required in very small amounts.
